# Current Literature

**Published:** 1882-02

**Authors:** 


### CURRENT LITERATURE.

Sir James Paget writes, in *Nineteenth Century*, on "Vivisection, its Pains and its Uses"

Dr. W. B. Carpenter gives, in *Nineteenth Century*, a good *résumé* of the present status of "Disease Germs," which has been copied into *Popular Science Monthly* and Littel's *Living Age*. Dr. C. tries to show that cow-pox and variola are one disease, differing only in intensity.

Mr. Henry Bergh writes, in *North American Review*, against vaccination. Some of his points are good; others, weak. He fears syphilis and scrofulous complaints; the one from the humanized virus; the other, from the animal virus.

Wm. Wood & Co. have added a *Monthly Supplement* to their *American Journal of Obstetrics*. The Supplement gives society reports, etc.; the Journal (a quarterly), long exhaustive articles.

H. C. Lea's Sons & Co. now publish their *Medical News* as a weekly.

